# Monitoring Circulating Tumor DNA During Surgical Treatment in Patients with Gastrointestinal Stromal Tumors

**DOI:** 10.1158/1535-7163.MCT-21-0403

**Published:** 2021-12-03

**Authors:** Gustav Johansson, Marta Berndsen, Stefan Lindskog, Tobias Österlund, Henrik Fagman, Andreas Muth, Anders Ståhlberg

**Affiliations:** 1 Sahlgrenska Center for Cancer Research, Department of Laboratory Medicine, Institute of Biomedicine, Sahlgrenska Academy, University of Gothenburg, Gothenburg, Sweden.; 2 Wallenberg Centre for Molecular and Translational Medicine, University of Gothenburg, Gothenburg, Sweden.; 3 Department of Surgery, Institute of Clinical Sciences, Sahlgrenska Academy, University of Gothenburg, Gothenburg, Sweden.; 4 Section of Endocrine and Sarcoma Surgery, Department of Surgery, Sahlgrenska University Hospital, Gothenburg, Sweden.; 5 Department of Surgery, Halland Regional Hospital Varberg, Region Halland, Varberg, Sweden.; 6 Region Västra Götaland, Department of Clinical Genetics and Genomics, Sahlgrenska University Hospital, Gothenburg, Sweden.; 7 Department of Clinical Pathology, Sahlgrenska University Hospital, Gothenburg, Sweden.

## Abstract

The majority of patients diagnosed with advanced gastrointestinal stromal tumors (GISTs) are successfully treated with a combination of surgery and tyrosine kinase inhibitors (TKIs). However, it remains challenging to monitor treatment efficacy and identify relapse early. Here, we utilized a sequencing strategy based on molecular barcodes and developed a GIST-specific panel to monitor tumor-specific and TKI resistance mutations in cell-free DNA and applied the approach to patients undergoing surgical treatment. Thirty-two patients with GISTs were included, and 161 blood plasma samples were collected and analyzed at routine visits before and after surgery and at the beginning, during, and after surgery. Patients were included regardless of their risk category. Our GIST-specific sequencing approach allowed detection of tumor-specific mutations and TKI resistance mutations with mutant allele frequency < 0.1%. Circulating tumor DNA (ctDNA) was detected in at least one timepoint in nine of 32 patients, ranging from 0.04% to 93% in mutant allele frequency. High-risk patients were more often ctDNA positive than other risk groups (*P* < 0.05). Patients with detectable ctDNA also displayed higher tumor cell proliferation rates (*P* < 0.01) and larger tumor sizes (*P* < 0.01). All patients who were ctDNA positive during surgery became negative after surgery. Finally, in two patients who progressed on TKI treatment, we detected multiple resistance mutations. Our data show that ctDNA may become a clinically useful biomarker in monitoring treatment efficacy in patients with high-risk GISTs and can assist in treatment decision making.

## Introduction

Gastrointestinal stromal tumor (GIST) is the most common abdominal sarcoma with a yearly incidence rate of 15 per 1,000,000 (1, 2). At diagnosis, 80% have a mutation in *KIT* and 10% in *PDGFRA*, resulting in tyrosine kinase activation and tumor cell proliferation (3–5). The clinical spectrum of GISTs ranges from incidentally discovered small tumors (< 2 cm) with low malignant potential (6) to highly malignant tumors with an aggressive clinical course (7). Risk stratification is based on tumor size, location, and mitotic count, according to the NIH consensus classification system (8–11). For patients with tumors in the very low-, low-, and intermediate-risk groups, surgical resection is often curative. For patients with high-risk tumors and treatment-sensitive mutations, tyrosine kinase inhibitors (TKIs), particularly imatinib, may be used both as neoadjuvant and adjuvant treatments (11–13). Neoadjuvant TKI treatment may facilitate surgery resulting in less morbidity (13), and TKI treatment has been shown to improve longtime survival (14). Secondary resistance with tumor progression within the first 2 years of treatment remains a significant clinical problem, affecting 40% to 50% of patients under TKI treatment for metastatic GISTs, usually due to resistance mutations occurring in either *KIT* or *PDGFRA* (5, 15, 16).

To date, monitoring treatment efficacy in patients diagnosed with GISTs relies on imaging, but sensitive and reliable liquid biopsy–based biomarkers to detect tumor progression and development of resistance mutations are lacking. Analysis of circulating tumor DNA (ctDNA) in plasma has become clinically relevant in the management of multiple cancer forms. It has been widely applied in screening, diagnostics, prognostics, monitoring treatment efficacy, early detection of treatment resistance, minimal residual disease, and relapse (17, 18). Cell-free DNA (cfDNA) may be released into the blood by apoptosis, necrosis, and secretion, while clearance occurs through nuclease activity, renal excretion, and uptake from spleen as well as liver (19, 20). The half-time of cfDNA, including ctDNA, is between 15 minutes and 2.5 hours (21). Hence, the ctDNA profile can be viewed as a real-time assessment of the patients' clinical status. Clinically relevant ctDNA levels often require the assessment of < 0.1% mutant allele frequencies. This analytic sensitivity can be achieved with methods, such as digital PCR and sequencing based on molecular barcodes (22). However, to our knowledge, no tailor-made strategy to monitor treatment efficacy and secondary resistance development in patients diagnosed with GISTs currently exist.

In this study, we developed a sequencing strategy based on simple, multiplexed, PCR-based barcoding of DNA for sensitive mutation detection using sequencing (SiMSen-Seq) (23, 24) that enables ctDNA analyses of both tumor-specific and TKI resistance mutations in patients diagnosed with GISTs at very low mutant allele frequency. We applied the approach to plasma samples collected from patients diagnosed with GISTs and correlated ctDNA levels to diagnostic and clinical parameters. Our tailor-made sequencing approach to analyze ctDNA opens new means to determine the clinical utility of ctDNA analysis in patients diagnosed with GISTs.

## Materials and Methods

### Patient inclusion and clinical assessments

All patients over 18 years diagnosed with GISTs from November 2016 to March 2019 (*n* = 210) at the Department of Surgery, Sahlgrenska University Hospital, Gothenburg, Sweden, were offered to be included in the study. Mutational analysis of *KIT*, *PDGFRA*, and *BRAF* was routinely performed as part of the diagnostic workup on either preoperative biopsies or resection specimens by targeted sequencing panels (Cancer Hotspot Panel v2 or Oncomine Focus, Thermo Fisher Scientific) at the Department of Clinical Pathology, Sahlgrenska University Hospital (Gothenburg, Sweden). The sampled cohort consisted of all risk groups, according to the updated NIH risk classification system (8) and at all disease stages, including those with metastases. The study group consisted of patients scheduled for surgery. Blood samples for ctDNA analysis were collected at routine clinical controls at 3- or 6-month intervals before and after surgery. Intraoperative samples were collected from an arterial line at the start of the surgery, when the tumor had been mobilized, and during wound closure. Data were retrieved from the medical records regarding age, gender, tumor location, tumor size, presence of metastases, length of neoadjuvant treatment, surgical procedure, the radicality of the surgery (R0/R1), and adjuvant treatment. Tumor size was measured either directly on the surgical specimen or by CT scan. After a scheduled interim analysis in September 2018, the study protocol was amended, and thereafter only patients with high-risk tumors were included. The study protocol and all amendments were approved by the regional ethical review board in Gothenburg, Sweden (No. 485-16, T795-16, and T525-18). Signed informed consent was obtained from each patient in accordance with Declaration of Helsinki.

### Sampling and extraction of cfDNA

Blood samples were collected in cf-DNA/cf-RNA Preservative Tubes (No. 63950, Norgen Biotek). Plasma was isolated 1 to 14 days after sampling by 20-minute single centrifugation, 430 g, at room temperature, using a high-speed swing-bucket 5804R centrifuge (Eppendorf). The plasma fraction (4–6 mL) was transferred to a 15 mL falcon tube (No. 62.554.502, Sarstedt) and stored at −80°C until cfDNA extraction.

Plasma was thawed in a water bath at room temperature and then immediately centrifuged for 10 minutes, using a fixed-angle rotor 5804R centrifuge, 16,000 × *g*, at 4°C. cfDNA was extracted from 4- to 6-mL plasma using Magmax cfDNA extraction kit (No. A29319, Thermo Fisher Scientific), according to manufacturer's instructions with a final elution volume of 75 μL but without initial protease treatment. Extracted cfDNA was concentrated using Vivacon 500 spin columns with 30,000 MWCO Hydrosart membrane (No. VN01H22, Sartorius), where the final volume was adjusted to 10 μL with the addition of nuclease-free water (No. 10977-035, Invitrogen). A subset of samples (Supplementary Table S1) was extracted using a QIAamp Circulating Nucleic Acid Kit (No. 55114, Qiagen), according to the manufacturer's instructions. These samples were concentrated using DNA Clean & Concentrator-5 (No. D4013, Zymo Research), followed by heat incubation at 95°C for 10 minutes in Thermomixer Compact block heater (Eppendorf). Extracted cfDNA was stored at −20°C until analysis.

### Sample quality controls

To monitor the performance of preanalytic steps, we included several quality controls before SiMSen-Seq analysis. Nonconcentrated cfDNA was quantified with Qubit Fluorometer version 3 using the Qubit dsDNA HS Assay Kit (No. Q33216, No. Q32851, both Invitrogen), according to the manufacturer's instructions. The degree of cellular DNA contamination (Supplementary Fig. S1A) was analyzed using quantitative PCR (qPCR) as described previously (25). Data analysis was performed using CFX maestro version 4.1 (Bio-Rad). The cycle of quantification values were determined by regression. A subset of samples was analyzed on a 7500 Fast Real-Time PCR system (Thermo Fisher Scientific) with the identical protocol, except that the reaction mix also contained 1× Reference Dye for Quantitative PCR (No. R4526, Sigma-Aldrich). These samples were analyzed with Thermo Fisher Connect online software (Thermo Fisher Scientific), and cycle of quantification values were determined using a threshold line. Nonfragmented human genomic DNA (No. 11691112001, Roche) was used as a reference to assess the degree of cellular DNA contamination. We considered more than 5% of cellular DNA as contamination.

After sample concentration, the amount of amplifiable cfDNA and PCR inhibition (Supplementary Fig. S1B) were assessed as described previously (25). cfDNA quantification was performed using a standard curve of human genomic DNA, ranging from 10 to 0.37 ng with threefold dilutions steps. Interplate calibrators were used to compensate for variations between qPCR runs. To avoid repeated freeze-thawing of cfDNA, the cellular DNA contamination test was performed simultaneously with the cfDNA quantification and inhibition test.

Finally, synthetic spike-in DNA controls (gBlocks, IDT) were added to a subset of samples (Supplementary Table S1) before library preparation to detect library construction failure (Supplementary Fig. S1C). These molecules were identical to the amplified sequence with an ATG trinucleotide insertion after the 3′-end of the forward primer (Supplementary Table S2). The spike-in control was quantified with Qubit Fluorometer version 3 using the Qubit dsDNA HS Assay Kit. The spike-in molecules were diluted and aliquoted using a buffer containing 1 μg/μL bovine serum albumin supplemented with 2.5% glycerol (No. B14, Thermo Fischer Scientific). The numbers of PDGFRA_18 spike-in molecules were compared between each samples where data between different sequencing experiments were median centered.

To determine whether a sample was undersequenced, the average number of sequence reads per unique molecular identifier was calculated after sequencing (Supplementary Fig. S1D). Samples were considered to be undersequenced if the average barcode family contained less than seven reads.

### Assay design and validation

Tumor-specific and treatment resistance SiMSen-Seq assays were designed according to published guidelines (23). In total, 13 assays were developed and used (Supplementary Table S3). Assays were validated both as individual assays and as different 5-plex assays. Supplementary Figure S2 shows a representative electropherogram for the most common 5-plex used.

### ctDNA analysis

ctDNA analysis was performed with SiMSen-Seq according to published guidelines using 2- to 4-μL concentrated cfDNA and 1-μL synthetic spike-in molecules (23). To maximize cfDNA load, the total volume of some reactions was increased by 50%, that is, 15-μL barcoding PCR, 30-μL inactivation buffer, and 60-μL adapter PCR. If an even higher volume were needed to include all extracted cfDNA, parallel reactions were performed and pooled after purification. Libraries were purified using Agencourt AMPure XP system (No. A63881, Beckman Coulter), according to the manufacturer's instructions using a 1:1 beads-to-sample ratio. Libraries were assessed using the HS NGS Fragment kit (No. DNF-474, Agilent) on a 5200 Fragment Analyzer System (Agilent) and quantified using the NEBNext Library Quant Kit (No. E7630, New England Biolabs).

Sequencing was performed on either MiniSeq using a High Output Reagent Kit (150 cycles, No. FC-420-1002, Illumina) or Nextseq using NextSeq 500/550 Mid Output Kit v2.5 (150 cycles, No. 20024907, Illumina). The final library concentrations were between 0.8 and 1.4 pmol/L, and 20% PhiX Control v3 (No. C-110-3001, Illumina) was used. Sequencing data were processed as described previously (23). Briefly, sequencing reads were aligned to the human genome from Genome Reference Consortium Human Build 38 (26). Reads with similar alignment and identical unique molecular identifier were grouped into families. A fixed threshold of at least three sequencing reads per unique molecular identifier family was used to construct consensus reads. Mutations were called after manual inspection of the binary alignment map file. Single-nucleotide variants required more than six consensus reads to be called. If variants with less than six consensus reads were detected, they were still considered variants if at least one other sample from the same patient displayed the same mutation with at least six consensus reads. Inserts and deletions were considered a variant with one consensus read only.

### Data analysis

Statistical calculations were performed using GraphPad prism version 8.4.3 (GraphPad Prism, RRID:SCR_002798). Figures were generated with either GraphPad prism or R version 4.0.1 (RRID:SCR_001905; ref. 27) using packages ggplot2 (RRID:SCR_014601; ref. 28) and ggpubr (RRID:SCR_021139; ref. 29). All sequencing data can be found in the Sequence Read Archive database with the accession number PRJNA749711. Processed data and supportive scripts are available at figshare (DOI:10.6084/m9.figshare.15059541).

## Results

### Description of the study group

**Figure 1.**
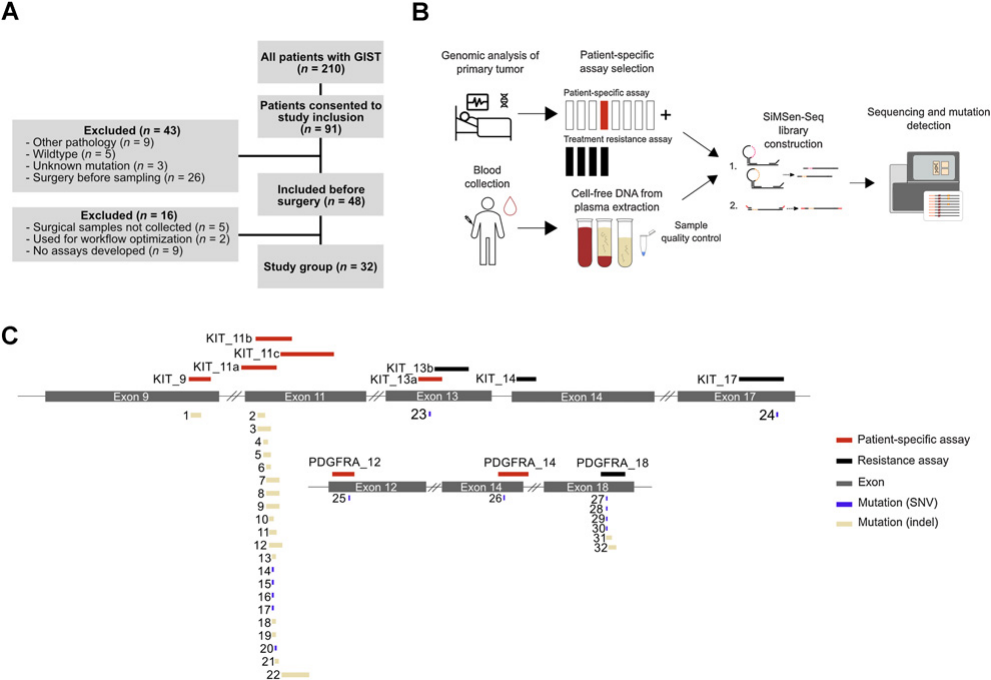
Experimental overview and primary tumor mutations. **A,** Consort diagram of the eligible patient cohort and study enrollment. The cohort consisted of all patients treated at the Deparment of Surgery, Sahlgrenska University Hospital, Gothenburg, Sweden, between November 2016 and March 2019. Forty-three of the 91 initially included patients were excluded. Of the excluded patients, nine patients revealed other diagnoses than GIST by pathology analysis, five patients displayed neither *KIT* nor *PDGFRA* mutations (wild-type) in tumor biopsy, three patients had no mutation analysis performed on the tumor material and 26 patients were enrolled after surgery and hence excluded. Thirty-two of 48 patients that were included before surgery were finally analyzed for the presence of ctDNA. Of the 16 additionally excluded patients, five patients were excluded, because sampling was not possible during surgery, samples from two patients were used in workflow optimization and nine patients were excluded as their tumor-specific mutations were not targeted by the developed assays. **B,** Five SiMSen-Seq assays were used to assess each patient, including one tumor-specific assay targeting the mutation identified in the tumor biopsy combined with four resistance assays. Blood samples were collected during routine visit before and after surgery. At surgery, samples were collected at start of surgery, during mobilization of the tumor and at closure. Extracted cfDNA was analyzed by SiMSen-Seq. Several quality controls were used to monitor the experimental performance. **C,** Assay overview and detected mutations in tumor biopsy. The length and exon position of each assay are shown. All types of mutations and their position are indicated for all 32 patients. SNV, single-nucleotide variant; indel, insertion or deletion mutation.

The overall study design and patient selection are shown in Fig. 1A. The study group consisted of 32 surgically treated patients. We classified seven patients as very low- or low-risk, six patients as intermediate-risk, and 19 patients as high-risk, according to the NIH risk classification system (8). Patient demographics and tumor details are described in Table 1. Six patients in the high-risk and one patient in the intermediate group had metastatic disease, of which four had synchronous and three had metachronous metastasis. In the tumor biopsy, 21 patients displayed primary mutations in *KIT* exon 11, while three patients had mutations in other *KIT* exons. Eight patients displayed mutations in *PDGFRA* exon 18.

**Table 1.** Patient demographics and tumor details.

	NIH risk group			
	Very low/Low	Intermediate	High	All
General
Number of patients	7	6	19	32
Sex (male/female)	2/5	2/4	9/10	13/19
Mean age (years)	63	69	61	63
ASA score (1:2/3)	2/5	1/5	7/12	10/22
Mean BMI	29	29	25	27
Tumor location				
Stomach	6	4	14	24
Small intestine	1	2	3	6
Esophagus	0	0	1	1
Rectum	0	0	1	1
Tumor properties				
Median tumor size (cm, range)	2 (1–4)	6 (5–10)	10 (3–23)	6 (1–23)
Median Ki-67 (%, range)	5 (1–10)	4.25 (1–15)	7.5 (1–60)	5 (1–60)
Localized/metastatic disease	7/0	5/1	13/6	25/7
*KIT* 11/*KIT* non-11^a^	3/0	3/1	15/2	21/3
*PDGFRA*^b^	4	2	2	8

Abbreviations: ASA, American Society of Anesthesiologists; BMI, body mass index.

^a^Primary mutations in *KIT* exon 11 (*KIT* 11) and in any exons except 11 (*KIT* non-11).

^b^Primary mutations in *PDGFRA*.

Neoadjuvant TKI treatment was given to 19 patients for 430 days on average, ranging from 106 to 989 days. Of these 19 patients, 13 were high-risk, while six patients were either intermediate-risk or low-risk, receiving neoadjuvant treatment to enable surgery with less morbidity, for example, gastric wedge resection instead of total gastrectomy. Surgical treatment details are summarized in Supplementary Table S4. A laparoscopic procedure was performed in 14 patients, whereas the remaining 18 patients underwent open procedures. Twenty-nine resections were classified as R0 and three as R1.

### Development of patient-specific SiMSen-Seq panels for patients diagnosed with GISTs

We developed patient-specific SiMSen-Seq panels that target the patient's tumor-specific mutation in either *KIT* or *PDGFRA*, as well as mutations related to imatinib and sunitinib resistance (Fig. 1B). The tumor-specific mutation was identified by sequencing the primary tumor, while the sequences for imatinib and sunitinib resistance in *KIT* and *PDGFRA* were identified from the COSMIC database (30). In total, we developed nine assays targeting tumor-specific mutations and four resistance assays (Fig. 1C). The resistance assays covered sequences, corresponding to 94% of all reported resistance cases (Supplementary Table S5). While the tumor-specific assay changed between patients, the four resistance assays were identical for all patient-specific SiMSen-Seq panels. All individual assays and 5-plex assays were evaluated on the basis of their efficiency and specificity (for details, see Materials and Methods). cfDNA, including ctDNA, is highly fragmented. Hence, all assays were designed to be short (65–99 bps) to increase their sensitivity (25).

**Figure 2.**
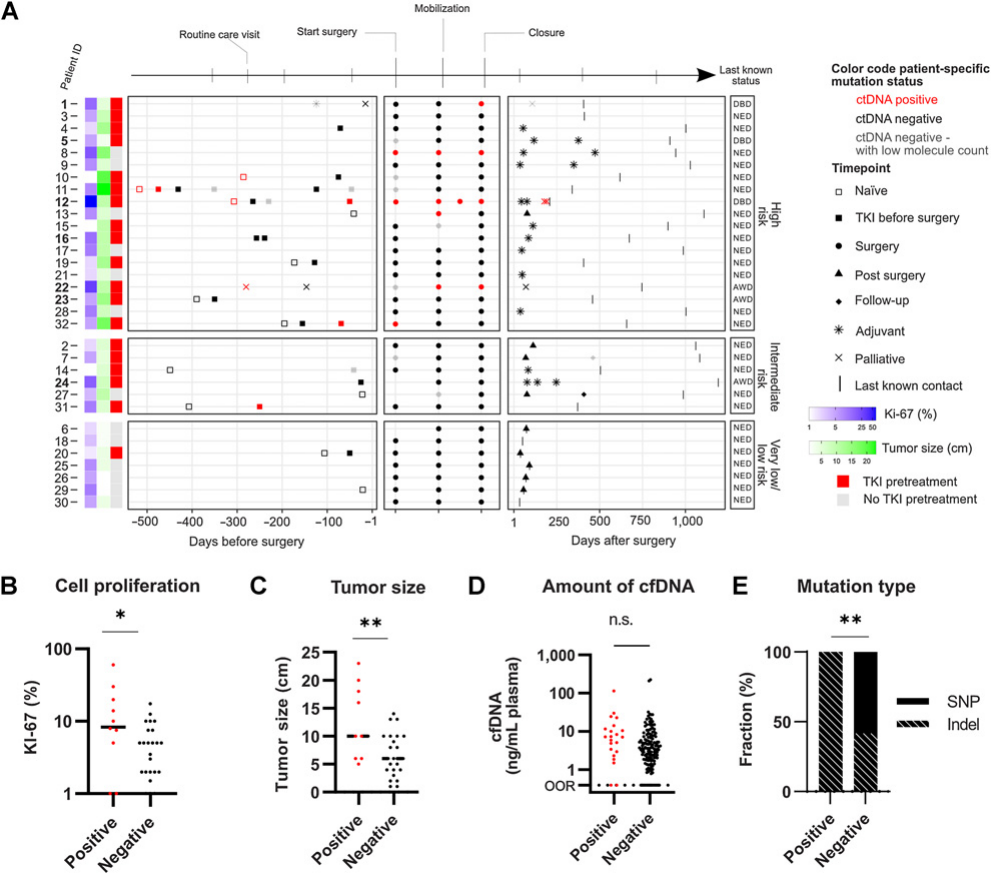
ctDNA characteristics in 32 patients diagnosed with GISTs. **A,** Detailed overview of tumor-specific ctDNA and clinical parameters. ctDNA status in relation to surgery is shown for each patient. Days at the bottom relates to surgical treatment. Red and black samples indicate samples being ctDNA positive and negative, respectively. Gray samples are negative samples, but with less than 50 cfDNA molecules detected. Patients with metastatic disease are shown as bold patient identification (ID). Information about Ki-67, tumor size, TKI treatment, and last known disease status is shown. NED, no evidence of disease; AWD, alive with disease; DBD, dead by disease. **B,** Tumor cell proliferation rates versus presence of ctDNA. Patients with at least one ctDNA-positive sample were considered positive. Data are shown in log_10_ scale. *n* = 32; *, *P* ≤ 0.05; Student *t* test on log-transformed values. **C,** Tumor size versus presence of ctDNA. Patients with at least one ctDNA-positive samples were considered positive. *n* = 32; **, *P* ≤ 0.01, Student *t* test. **D,** Total ctDNA levels versus presence of ctDNA in each sample. Data are shown in log_10_ scale. Values out of range (OOR) was in statistical calculation replaced with the value 0.39, which is the lowest detected value divided by two, *n* = 161. n.s., not significant, Student *t* test on log-transformed values. **E,** Type of tumor-specific mutation detected as ctDNA. Frequency of single-nucleotide variations (SNP) and indel. *n* = 32; **, *P* ≤ 0.01; Fisher exact test.

**Figure 3.**
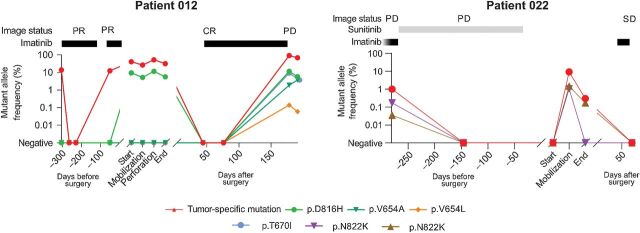
Disease status in relation to ctDNA levels of tumor-specific and TKI resistance mutations. Black and gray bars indicate treatment duration. Disease status according to imaging. CR, complete response; PD, progressive disease; PR, partial response; SD, stable disease.

In total, we analyzed 161 plasma samples. SiMSen-Seq failed to detect any molecules in one sample, and 11 samples resulted in < 50 detected cfDNA molecules. Three of 111 samples were contaminated with more than 5% cellular DNA (Supplementary Fig. S1A), 15 and six of 161 samples were inhibited or strongly inhibited, respectively (Supplementary Fig. S1B). Sixteen and 10 of 152 samples displayed lower and higher amounts of spike-in molecules than expected, respectively (Supplementary Fig. S1C). Sixteen of 155 samples were undersequenced, defined as a mean of less than seven sequence reads per unique molecule identifier (Supplementary Fig. S1D). The mean cfDNA concentration of all plasma samples was 9.26 ng/mL plasma, where surgical samples displayed significantly higher cfDNA concentrations (Supplementary Fig. S3). No differences in cfDNA concentrations were observed when comparing high-risk patients with the combined intermediate- and low-risk group for any sampling timepoints.

### Detection of ctDNA correlated to clinical characteristics

During the study period, including surgery and follow-up, the tumor-specific mutation was detected as ctDNA in nine of 32 patients in at least one sample (Fig. 2A; Supplementary Table S6), ranging from 0.04% to 93% mutant allele frequency. Furthermore, ctDNA was detected in 22 of 161 plasma samples, primarily during surgery (Table 2) and during progressive and stable disease determined by imaging (Supplementary Table S7). High-risk patients were overrepresented compared with the other risk groups combined (Table 2). Accordingly, patients with detectable ctDNA at any sample timepoint displayed a significantly higher tumor cell proliferation rates, shown by Ki-67 index staining and larger tumor sizes (Fig. 2B and C). However, the total cfDNA levels were not elevated in ctDNA-positive samples (Fig. 2D). Nine tumor-specific mutations were detected in ctDNA, one in *KIT* exon 9, six in *KIT* exon 11, and two in *PDGFRA* exon 18, all mutations were insertions or deletions, that is, indels (Fig. 2E).

**Table 2.** Number of patients with tumor-specific mutations detected in cfDNA.

	Presurgery	Surgery	Postsurgery	Any time
High-risk (*n* = 19)
Negative	6	13	12	11
Positive	5	6	1	8^a^
Intermediate (*n* = 6)				
Negative	3	6	5	5
Positive	1	0	0	1
Low-risk (*n* = 7)				
Negative	2	7	5	7
Positive	0	0	0	0
All (*n* = 32)				
Negative	11	26	22	23
Positive	6	6	1	9

^a^Significant compared with other risk groups using Fisher exact test, *P* < 0.05%.

ctDNA was detected in four patients before the initiation of neoadjuvant TKI treatment or at the first neoadjuvant treatment control. In all cases, the ctDNA levels decreased or disappeared in the samples collected after initiated treatments and tumor regressions were observed by imaging (Fig. 2A). During surgery, six patients were ctDNA positive, and all of these patients were classified as high-risk for recurrence. In the three patients with nonradical resections (R1), ctDNA was detected in the surgical plasma samples collected at wound closure (patients 1, 8, and 12). All patients who were ctDNA positive during surgery became ctDNA negative in the first follow-up sample approximately 4 weeks after surgery. Patients with detectable ctDNA had a tendency toward larger tumor sizes (median 17 vs. 9 cm) and higher Ki-67 index (median 8 vs. 5%), none were statistically significant. In naïve samples, the ctDNA test sensitivity was 25% (43% for high-risk only). The ctDNA test displayed a sensitivity of 24% and specificity of 100%, when comparing imaging and ctDNA analysis (Supplementary Table S7). We assumed that complete response should be ctDNA negative, while progressive, regressive, and stable disease should be ctDNA positive.

Seven patients had metastatic disease, but only three were ctDNA positive in at least one sample. In these three patients, detection of ctDNA was associated with disease progression. Of the four ctDNA-negative patients, three were included in the study after initiation of TKI treatment and were responding. In the last of the four negative patients (patient 23), a detailed analysis of the treatment-naïve sample showed that the tumor-specific mutation was detectable but below the applied threshold to be ctDNA positive.

Two patients, 20 and 4, had no tumor-specific mutation detected in cfDNA. Instead, a secondary mutation in *KIT* exon 11 p.G565E (COSV55392044) was observed. The mutation was detected in all samples for patient 20, with a mutant allele frequency between 0.08% and 2.7%. The mutation was only detected in the last sample for patient 4 with a mutant allele frequency equal to 3.3%. This mutation is previously not reported in GIST, but is annotated as pathogenic in COSMIC using functional analysis through hidden Markov models (30). Because of the unknown relevance of this mutation, it was not included in downstream analyses.

### Clinical characteristics of patients with detectable resistance mutations

Mutations known to cause TKI resistance in GISTs were detected in patients 12 and 22. Patient 12 had a 10-cm rectal GIST with a mitotic count > 10 mitoses per 5 mm^2^ and a *KIT* exon 11 mutation in the tumor biopsy (Fig. 3). The treatment-naïve sample showed ctDNA that disappeared after neoadjuvant imatinib treatment, corresponding to tumor response at imaging. The imatinib treatment was paused due to suspected drug intolerance, and ctDNA was detected again after 8 weeks. During the rectal amputation procedure, the ctDNA frequency was 25%, with a peak of 52% following a rupture of the tumor capsule. A treatment resistance mutation was detected in all surgical samples, but at lower frequencies (5%–12%). Imatinib treatment was reinitiated 6 weeks after surgery, and no ctDNA was observed 5 and 10 weeks after surgery, in line with imaging showing complete response. However, 6 months postoperatively, ctDNA increased to > 90% as the patient was admitted with a disseminated disease burden in the pelvis and shortly thereafter died. Now, three additional known and eight previously unannotated resistance mutations were detected (Supplementary Table S1).

Patient 22 had a 10-cm duodenal GIST and liver metastases. The tumor biopsy from the duodenum showed a mutation in *KIT* exon 11. In the first neoadjuvant sample following 19 months of imatinib treatment, we detected the tumor-specific mutation and two resistance mutations. Because of primary tumor progression, although the liver metastases showed regress, the treatment strategy was changed to second-line TKI, sunitinib. Thereafter, we detected neither the tumor-specific mutation nor the resistance mutations. As palliative duodenal resection was scheduled, no TKI treatment was given 1 month prior to the procedure. No cfDNA and hence, no ctDNA was detected in the sample collected at the start of the surgery. However, at wound closure, the tumor-specific as well as the two resistance mutations reoccurred. Two months after surgery, as imatinib had been reinitiated, no further growth of the liver metastases was observed by imaging, and no ctDNA was detected.

## Discussion

We developed a GIST-specific sequencing approach to monitor treatment efficacy by analyzing both the patients' tumor-specific mutation and sequences related to TKI resistance. Using SiMSen-Seq, we analyzed ctDNA before, during, and after surgical treatment in an observational study including patients with GIST from all risk groups. SiMSen-Seq uses unique molecular identifiers that remove sequencing errors and correct for amplification biases, enabling reliable ctDNA detection at mutant allele frequencies < 0.1% (23). We detected ctDNA in 28% of the studied patients, primarily in high-risk patients with large tumors and high proliferation rates. In 32% of high-risk patients, we detected ctDNA intraoperatively, where half had received neoadjuvant TKI treatment. In neoadjuvant TKI-treated patients, ctDNA levels decreased or disappeared during treatment, which correlated with radiological tumor response. In patients with metastatic disease, ctDNA was detected in patients with progressive disease. Furthermore, we detected secondary TKI resistance mutations in two patients who displayed tumor progression.

Other studies have reported detectable ctDNA in the range of 29% to 72% (31–33). These studies and our included small and heterogeneous patient cohorts, but all were consistent in detecting ctDNA primarily in advanced GISTs. The discrepancy in detection rates may be explained by several factors, such as inclusions of different risk groups, treatment status, and disease activity. For example, a recent study reported a ctDNA detection rate of 92% in a cohort of 25 patients, where most displayed metastatic and active disease at inclusion (34). Earlier studies from the same research group detected ctDNA in 50% of patients with active disease (18 patients) and in 39% of patients with both active disease and complete remission (38 patients; ref. 31). Here, all but one patient were classified as high-risk. The prognosis of patients with GIST is dependent on the mitotic rate, tumor location, and tumor size (8). We found that the presence of ctDNA correlated with both high tumor cell proliferation rates, shown by Ki-67 staining, and large tumor size, which are in agreement with other studies (33–36). Risk categorization of patients with neoadjuvant TKI treatment is a clinical challenge due to the difficulties of assessing the mitotic count in a TKI-treated specimen. Patient 31 had a 6-cm large tumor, and the mitotic count was not assessable due to neoadjuvant TKI treatment. The patient was categorized as intermediate risk of recurrence after a clinical discussion. The Ki-67 index in the tumor specimen was 10%, and the patient had detectable ctDNA in one sample before surgery (Fig. 2A). If an alternative GIST risk score based on tumor size and Ki-67 index had been applied, the patient would instead be categorized in the high-risk group (1).

To our knowledge, this is the first time intraoperative ctDNA dynamics have been assessed in GISTs. The observation that none of the patients with either low- or very low-risk tumors showed detectable ctDNA in any samples, not even during mobilization in surgical treatment, indicates a more benign nature compared with high-risk tumors, and this is also supported by previously published data (31, 32, 36). All high-risk patients with detectable ctDNA during surgical treatment displayed a complete response with no detectable tumor-specific ctDNA 4 weeks following surgery. Although there is a variation of ctDNA shedding intraoperatively among patients with high-risk GIST tumors, all patients with nonradical surgery, known to correlate with poor prognosis (37, 38), were ctDNA positive. Our and other data (33) show that tumor-specific mutations that occur as insertions or deletions are more likely to be ctDNA positive. According to a randomized clinical trial, Scandinavian Sarcoma Group XVIII, patients with *KIT* exon 11 insertions or deletions have an unfavorable recurrence-free survival compared with patients with other mutations (39). In a recent study, ctDNA was detected in 13 of 14 metastasized patients at the start or the change of TKI treatment (40). In agreement with our study, six of nine patients were ctDNA negative 6 weeks after the start of TKI treatment. Hence, ctDNA may be a useful biomarker detecting progressive metastatic GIST disease.

Detection of TKI resistance is challenging. Current methods, such as imaging, have limited sensitivity and do not reveal any molecular information. Additional tissue biopsies are often not feasible to collect due to technical and clinical issues, such as the risk of missing clonality due to tumor heterogeneity and complications related to biopsy collection (41, 42). We detected secondary resistance mutations in two patients, which could have affected the TKI treatment strategy for these patients if known. Interestingly, we detected a secondary *KIT* exon 11 (G565E) mutation in two other patients, but no tumor-specific mutation. This mutation is previously not reported in GIST but in a patient diagnosed with melanoma, who also displayed a *KIT* W557R mutation (43). Our mutation analyses did not include any matched normal sample for comparison. Hence, one possibility is that this mutation occurred in a subpopulation of nontumor cells (44).

Cross-sectional imaging is the golden standard for staging and follow-up of GIST, but may benefit from complementary liquid biopsy analysis. Profiling of ctDNA may provide increased sensitivity to detect disease and relapse early, optimized timing of imaging after surgery, and molecular information related to TKI treatment strategy. A limitation of the present proof-of-concept study is the limited number of patients and samples. Multicenter studies of larger patient cohorts are needed to determine true clinical utility, where prospective studies may focus on high-risk patients aiming at identifying patients with relapse and TKI resistance before clinical onset. A potential limitation with our patient-specific sequencing strategy and other targeted approaches is that *de novo* secondary mutations beyond the included sequences related to TKI resistance cannot be identified. Furthermore, accurate ctDNA analysis requires the entire workflow to be optimized. To assess the performance of cfDNA extraction and sequencing, we applied several quality control and metrics. On the basis of our data, we could not detect any systematic biases in the developed experimental workflow.

In summary, we have developed a GIST-specific sequencing approach to assess ctDNA in an optimized experimental workflow and report ctDNA dynamics in relation to surgery. Our analyses reveal potential prognostic ctDNA data that may facilitate the management of patients with high-risk GIST.

## Authors’ Disclosures

G. Johansson reports grants from Assar Gabrielssons Research Foundation, Johan Jansson Foundation for Cancer Research, and Anna-Lisa och Bror Björnsson stiftelse during the conduct of the study; personal fees and other support from Simsen Diagnostics; and personal fees from AstraZeneca outside the submitted work. S. Lindskog reports grants from Johan Jansson Foundation and Gothenburg Medical Association during the conduct of the study. T. Österlund reports grants from Region Västra Götaland, Sweden, Assar Gabrielsson Foundation, and Johan Jansson Foundation during the conduct of the study and reports employment with Sahlgrenska University Hospital, Gothenburg, Sweden and University of Gothenburg, Gothenburg, Sweden. A. Ståhlberg reports grants from Region Västra Götaland, Sweden, Swedish Cancer Society (19-0306), Swedish Research Council (2017-01392), the Swedish state under the agreement between the Swedish government and the county councils, the ALF-agreement (716321), and Sweden's Innovation Agency (2018-00421 and 2020-04141) during the conduct of the study; other support from TATAA Biocenter, SiMSen Diagnostics, and Iscaff Pharma outside the submitted work; in addition, A. Ståhlberg has a patent for Protection of barcodes during DNA amplification using molecular hairpins, US10557134B2 issued to Boston University and Ontario Institute for Cancers Research; and reports employment with Sahlgrenska University Hospital, Gothenburg, Sweden and University of Gothenburg, Gothenburg, Sweden. No disclosures were reported by the other authors.

## Supplementary Material

Supplementary Figures Supplementary Figure S1 shows assessment of quality controls when analyzing cfDNA. Supplementary Figure S2 shows a representative electropherogram of assay performance Supplementary Figure S3 shows a the amount of cfDNA in plasma for different risk groups and sample types.

Supplementary Tables Supplementary Table S1 shows detailed sample characteristics. Supplementary Table S2 shows sequences of synthetic spike-in DNA. Supplementary Table S3 shows assay sequences. Supplementary Table S4 shows details related to surgical treatment. Supplementary Table S5 shows resistance events of tyrosine kinase inhibitors in the COSMIC database. Supplementary Table S6 shows patient characteristics. Supplementary Table S7 shows concordance between imaging and ctDNA.
