# Supplementary material for: Monitoring Circulating Tumor DNA During Surgical Treatment in Patients with Gastrointestinal Stromal Tumors
Source: Mol Cancer Ther. 2021 Dec 3;20(12):2568–76. doi: 10.1158/1535-7163.MCT-21-0403 (PMC9398151; doi:10.1158/1535-7163.MCT-21-0403)
Supplement: Supplementary Figures — Supplementary Figure S1 shows assessment of quality controls when analyzing cfDNA. Supplementary Figure S2 shows a representative electropherogram of assay performance Supplementary Figure S3 shows a the amount of cfDNA in plasma for different risk groups and sample types. [file 15357163mct210403-sup-264922_2_supp_7338540_qy3h8n.pdf]

Supplementary Figures

Monitoring circulating tumor-DNA during surgical treatment in patients with gastrointestinal stromal tumors

Gustav Johansson[ab#], Marta Berndsen[cd#], Stefan Lindskog[cdf], Tobias Österlund[be], Henrik Fagman[ag], Andreas Muth[cd*], Anders Ståhlberg[abe*]

[a]Sahlgrenska Center for Cancer Research, Department of Laboratory Medicine, Institute of Biomedicine, Sahlgrenska Academy at University of Gothenburg, Gothenburg, Sweden.
[b]Wallenberg Centre for Molecular and Translational Medicine, University of Gothenburg, Gothenburg, Sweden.
[c]Department of Surgery, Institute of Clinical Sciences, Sahlgrenska Academy at University of Gothenburg, Gothenburg, Sweden
[d]Section of Endocrine and Sarcoma Surgery, Department of Surgery, Sahlgrenska University Hospital, Gothenburg, Sweden
[e]Region Västra Götaland, Sahlgrenska University Hospital, Department of Clinical Genetics and Genomics, Gothenburg, Sweden
[f]Department of Surgery, Halland Regional Hospital Varberg, Region Halland, Varberg, Sweden
[g]Department of Clinical Pathology, Sahlgrenska University Hospital, Gothenburg, Sweden.

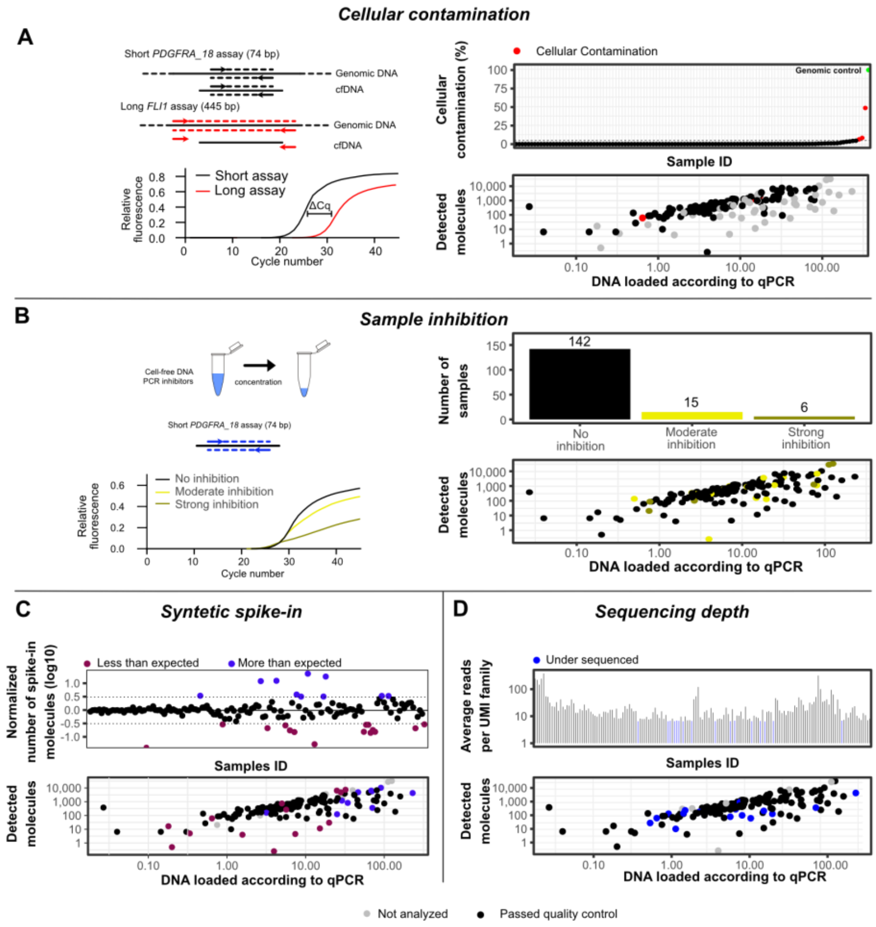

Supplementary Figure S1. Assessment of quality controls analyzing cfDNA. (A) Cellular contamination. Two assays, one short and one long qPCR assay were used to quantify fragmented and non-fragmented DNA, respectively. A sample with 5% cellular DNA was considered contaminated. Right top panel show cellular contaminations in all samples, sorted from lowest to highest cellular contamination. Right bottom panel shows the relation between samples with cellular contamination compared to the number of detected molecules from sequencing in the assay targeting the tumor-specific mutation versus the amount of DNA loaded into SiMSen-Seq. (B) Detection of PCR inhibition. Samples were concentrated and then tested for inhibition by qPCR. The shape of the amplification curve was used to assess the degree of inhibition. Samples were scored as no inhibition, moderate inhibition, and strong inhibition. Right top panel shows a summary of inhibited samples. Right bottom panel shows the relation between inhibited samples compared to the number of detected molecules from sequencing in the assay targeting the tumor-specific mutation versus the amount of DNA loaded into SiMSen-Seq. (C) Synthetic spike-in DNA controls. For each sequencing experiment, the same amount of synthetic spike-in DNA was added to each sample. Top panel shows the number of detected spike-in molecules normalized to the median within each sequencing round. An arbitrarily cut-off is shown as dotted line. Bottom panel shows the relation between samples with spike-in DNA compared to the number of detected molecules from sequencing in the assay targeting the tumor-specific mutation versus the amount of DNA loaded into SiMSen-Seq. (D) Evaluation of sequencing depth. Top panel shows the average number of reads within a UMI family as an average calculated from all assays analyzed per sample. Samples with average family size < 7 reads tumor-specific mutation versus the amount of DNA loaded into SiMSen-Seq. Bottom panel shows the relation between samples with different sequencing depths compared to the number of detected molecules from sequencing in the assay targeting the tumor-specific mutation versus the amount of DNA loaded into SiMSen-Seq.

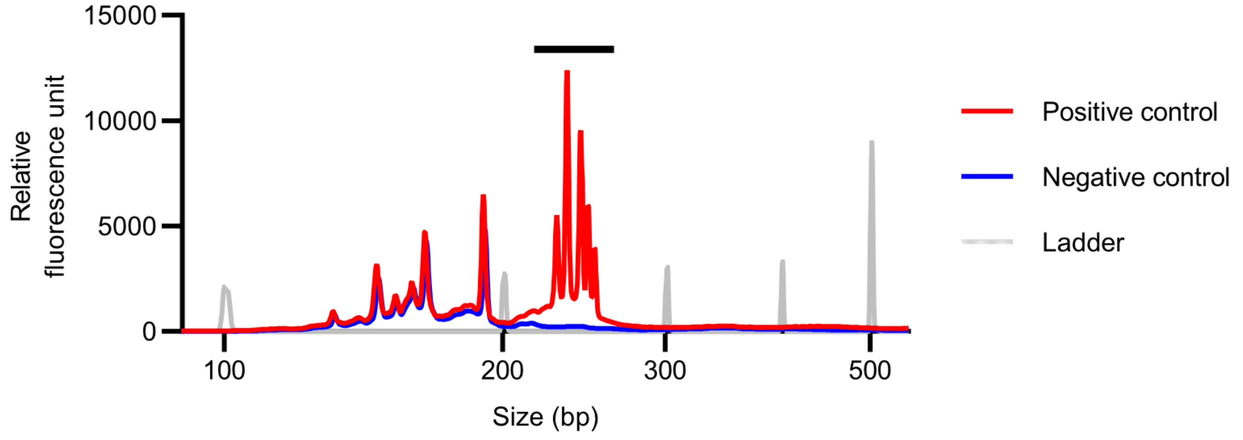

Supplementary Figure S2. Example of assay performance. Representative electropherogram of a 5-plex panel analyzed by parallel capillary electrophoresis using 20 ng genomic DNA (red). The black line above the electropherogram indicates correct PCR products. Negative water control in SiMSen-Seq (blue) and ladder (gray) are also shown.

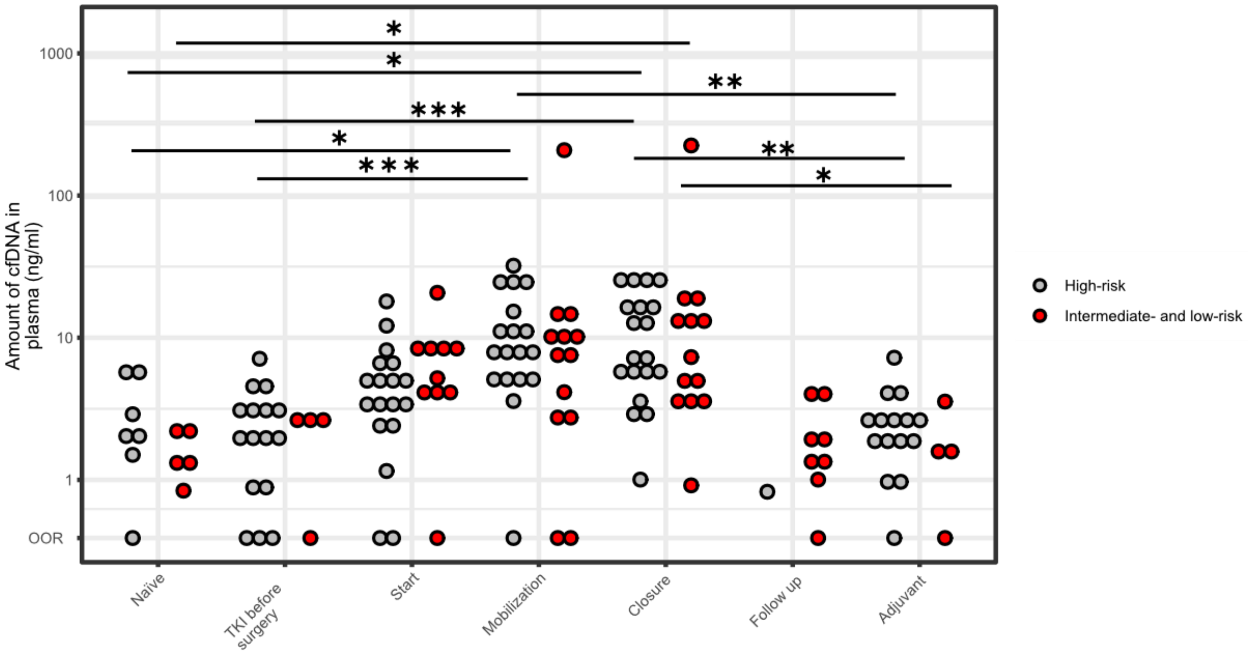

Supplementary Figure S3. Amount of cfDNA in plasma for different risk groups and sample types. Sample types and risk factors were compared using two-way ANOVA using log transformed values. Pairwise comparisons within each risk groups was performed with Turkey's multiple comparison test. The follow-up time point was excluded from statistical analysis, since the high-risk group only contained one sample. Values out of range (OOR) was replaced with the value 0.39, which is the lowest detected value divided by two * P ≤ 0.05, ** P ≤ 0.01, *** P ≤ 0.001.
